# Efficacy of psychosocial interventions to reduce affective symptoms in sexual and gender minorities: a systematic review and meta-analysis of randomized controlled trials

**DOI:** 10.1186/s12888-023-05451-y

**Published:** 2024-01-02

**Authors:** Yawen Yang, Zhiyu Ye, Wentian Li, Ye Sun, Lisha Dai

**Affiliations:** 1 Research Center for Psychological and Health Sciences, China University of Geosciences, Wuhan, Wuhan, Hubei Province 430074 China; 2 Psychosomatic Department, Wuhan Mental Health Center, Wuhan, China; 3 Psychosomatic Department, Wuhan Hospital for Psychotherapy, Wuhan, China

**Keywords:** LGBT, Sexual and gender minorities, Affective symptoms, Psychosocial interventions, Systematic review, Meta-analysis

## Abstract

**Background:**

Lesbian, gay, bisexual, transgender, and queer/questioning (LGBTQ) individuals are more likely than cisgender heterosexuals to experience mental, physical, and sexual health issues. A promising contemporary strategy to address the issue of affective symptoms in sexual and gender minorities (SGM) is psychosocial intervention.

**Objective:**

To systematically evaluate the effect of psychosocial interventions on the improvement of affective symptoms in SGM, and to provide a reference for the implementation of effective psychological interventions for SGM with affective symptoms.

**Methods:**

Between the date of database construction until December 10, 2022, a computerized search of the English-language literature published both nationally and worldwide was done. 8 literature databases and 3 additional gray databases were searched. We gathered randomized controlled trials that used psychological interventions for SGM. To evaluate risk bias in included papers in accordance with Cochrane cooperation criteria, we used Review Manager 5.4 software. In conjunction with post-test and follow-up data, mean differences were standardized using Stata 12.0 software. Subgroup analysis was used to investigate the cause of heterogeneity. The study was conducted strictly in accordance with PRISMA guidelines, and it was registered on the PROSPERO platform (CRD42023408610).

**Results:**

This review covered 18 research, and 14 studies were included in the meta-analysis. A total of 1194 study cases, including 706 cases from the control group and 488 cases from the experimental group, were included in these investigations. Compared to the control group, the psychosocial intervention group had significantly lower levels of depression (standardized mean difference (SMD) = -0.17;95% CI = [-0.30, -0.04]; *p* = 0.012) and anxiety (SMD = -0.22; 95% CI = [-0.41, -0.04]; *p* = 0.01), but no significant differences were found for distress (SMD = -0.19; 95% CI = [-0.45,0.07]; *p* = 0.021).

**Conclusion:**

According to this study, psychosocial interventions helped lessen the symptoms of depression and anxiety in SGM but had no significant effect on their psychological distress. To assess the impact of psychological intervention on SGM, more randomized controlled trials with larger sample sizes and numerous follow-up times should be done.

**Supplementary Information:**

The online version contains supplementary material available at 10.1186/s12888-023-05451-y.

## Introduction

A sexual minority is a person whose sexual orientation differs from the majority of the surrounding society, and can include gay, lesbian, bisexual and queer/questioning identities. Transgender people have a gender identity that differs from their sex assigned at birth, whereas cisgender people have a gender identity or gender expression that matches their sex assigned at birth. Sexual orientation is a multidimensional construct made up of at least three dimensions: sexual identity, attractions to the same or other sexes, and sex/gender of sexual partners [1, 2]. In spite of the increasing recognition of sexual and gender minority (SGM) populations, researches indicate that they face heightened psychological vulnerabilities in comparison to heterosexual individuals. These vulnerabilities encompass affective symptoms, such as depression [3], anxiety, distress [4] or other emotional status [5], non-suicidal self-injury [6], suicide [7] and others. As shown by the findings of the Youth Risk Behavior Survey conducted by the Centers for Disease Control and Prevention, it was observed that sexual minority youths had a much higher likelihood of engaging in suicidal behaviors compared to their heterosexual counterparts, with a prevalence rate above 60% [8]. A meta-analysis showed that sexual minority youth were three times more likely to have depression or depressive symptoms than their peers [9] Similarly, a national survey in the United States found that sexual and gender minority college students (*N* = 72,815) were twice as likely to report severe functioning-impairing depression and three times as likely to have suicidal thoughts compared to their heterosexual and non-transgender peers [10]. The minority stress hypothesis contends that discrimination experienced by individuals can lead to chronic stress and resultant medical and mental health problems [11].

Fortunately, a significant amount of research exists about the treatment of widespread mental health issues, such as depression and anxiety, particularly among the younger population. The National Institute for Health and Care Excellence (NICE) treatment recommendation in the United Kingdom [12] recommends the use of cognitive behavioral therapy (CBT) for children and young individuals with moderate to severe depression. However, prior research has shown worse clinical results among individuals identifying as SGM who undergo mainstream therapy compared to their heterosexual cisgender peers [13, 14]. The disparity is particularly evident among lesbian and bisexual adults [14] and gender minority adolescents [13] It was concluded that LGBT people are more likely to experience health inequalities due to heteronormativity or heterosexism, experiences of minority pressure, victimization and discrimination, and stigma [15]. In light of the perceived inefficacy and limited acceptance of mainstream services for SGM, it is imperative to undertake an examination of the therapeutic viability and use of broader SGM support systems. However, it is worth noting that there have been just 24 treatments specifically targeting SGM populations identified on a worldwide scale. The majority of these interventions (*n* = 17) primarily address sexual health concerns [16].

So far, researchers have diligently investigated psychological therapies for SGM. Several systematic reviews have been conducted on intervention for SGM. In a review conducted by Chaudoir et al. in 2017, a total of 44 interventions were identified that have the potential to alleviate sexual minority stress. These interventions encompass a wide range of areas, including education, mental and medical health care delivery, parent–child relationships, policy development and implementation, as well as role-playing activities and didactic lectures. The study revealed that the majority of therapies focused on directly mitigating stresses experienced by sexual minorities, rather than bolstering their coping mechanisms. Additionally, it was observed that a significant proportion (38; 86.4%) of these interventions were still in the preliminary phases of effectiveness testing and did not include a control group [17]. One study also observed that psychological and behavioral therapies that fail to acknowledge and address the stigma encountered by those identifying as sexual and gender minorities may exhibit reduced effectiveness [18]. In their scoping study, Lucassen et al. emphasized the significance and adaptability of digital interventions within the context of treatments targeting sexual and gender minority populations [19]. Moradi and Budge conducted a review that examined the topic of LGBQ + positive psychotherapy [20]. In conclusion, the aforementioned studies have encapsulated several intervention strategies for SGM and have offered distinct perspectives. However, a thorough and systematic evaluation of the effectiveness of psychosocial therapies on affective symptoms in SGM individuals has yet to be conducted. When considering the available data, it is well acknowledged that systematic reviews and meta-analyses are often regarded as the most dependable sources [21]. The existing literature on psychological therapeutic approaches for SGM has a limited number of reviews that include meta-analyses. Consequently, it is imperative to further research in this area by using quantitative research methodologies and integrating data from randomized controlled trials.

The purpose of this study was to collect randomized controlled trials of psychosocial interventions for SGM and to evaluate the efficacy of psychosocial interventions on affective symptoms in SGM using meta-analysis. Numerous prior investigations have been conducted to explore diverse therapeutic strategies targeting for SGM. In a study conducted by Craig et al. [22], an affirmative cognitive behavioral group treatment was provided to SGM adolescents and young adults in a community setting in Ontario, Canada. The findings revealed a noteworthy decrease in depressive symptoms among SGM individuals who received psychosocial interventions, as compared to those on the waitlist. In their study, Pachankis et al. [23] employed a concise online intervention to examine the impact on the psychological and behavioral well-being of LGBTQ youth. The findings revealed a greater decrease in depressive symptoms among participants in the expressive writing group SGM, as compared to those in the control group. However, it is worth noting that the reductions in anxiety and distress symptoms did not reach statistical significance. In their study, Carrico et al. [24] used cognitive behavioral stress management techniques, which were shown to be ineffective in alleviating depression symptoms among individuals in the homosexual men living with HIV. The objective of this study is to synthesize the outcomes of diverse intervention strategies in order to assess the effectiveness of psychosocial interventions on affective symptoms in SGM. Additionally, subgroup analysis was conducted to investigate the variations in efficacy among different types, formats, forms, and durations of interventions. By doing so, this research aims to generate empirical evidence that can enhance the promotion of psychosocial interventions in SGM.

Table 1 describes key terms used in this article.

**Table 1:** Key Terms

SGM	Sexual and gender minority
LGBTQ	Lesbian, gay, bisexual, transgender, and queer/questioning
NICE	The National Institute for Health and Care Excellence
SGMY	Sexual and gender minority youth
CBT	Cognitive behavioral therapy
CBSM	Cognitive-behavioral stress management
AFFIRM	The affirmative CBT group intervention
ESTEEM	Effective Skills to Empower Effective Men
EQuIP	Empowering Queer Identities in Psychotherapy
CBT-BISC	Cognitive Behavioral Therapy for Body Image and Self-Care
HAART	Highly active anti-retroviral therapy
MBSR	Mindfulness-based stress reduction
ES-HIM	Enhanced Sexual Health Intervention for Men

## Method

This study was conducted in strict accordance with the PRISMA (Preferred Reporting Items for Systematic Reviews and Meta-Analyses) [25] specifications and was registered with PROSPERO (International Prospective Register of Systematic Reviews) with the registration number CRD42023408610.

### Search strategy

Systematic searches were performed on computerized databases to retrieve English-language material published up to December 10, 2022. Eight literature databases were searched, including PubMed, Embase, Cochrane Library, CNKI, Wanfang Data, Web of Science, PsycINFO (APA PsycNet), ClinicalTrials.gov, and 3 other gray databases: MedRxiv, ChinaXiv, SSRN. Subject terms used for the search included sexual and gender minorities, psychosocial interventions, and randomized controlled trials. The topic term of each database remains consistent, but modified to adhere to the specific needs of each database operator. The search formulas for each database can be found in Appendix 1. The full PubMed search formula is ((((("Psychosocial Intervention"[Mesh]) OR ((psychosocial intervention*[Title/Abstract]) OR ( psychological intervention*[Title/Abstract])) OR (("Psychotherapy"[Mesh]) OR (psychotherap*[Title/Abstract])) OR (psychoeducation[Title /Abstract])) AND (("Sexual and Gender Minorities"[Mesh]) OR ((((((((((((((((((Non?Heterosexual*[Title/Abstract]) OR (Sexual Dissident*[Title /Abstract])) OR (GLBT Person*[Title/Abstract])) OR (GLBTQ Person*[Title/Abstract])) OR (LGBT Person*[Title/Abstract])) OR (LGBTQ Person*[Title /Abstract])) OR (Lesbigay Person*[Title/Abstract])) OR (Non?Heterosexual Person*[Title/Abstract])) OR (Sexual Minorit*[Title/Abstract])) OR ( LBG Person*[Title/Abstract])) OR (Gay*[Title/Abstract])) OR (Men Who Have Sex With Men[Title/Abstract])) OR (Gender Minorit*[Title/Abstract])) OR (Lesbian*[Title/Abstract])) OR (Women Who Have Sex With Women[Title/Abstract])) OR (Bisexual*[Title/Abstract])) OR (Homosexual*[Title/Abstract])) OR (Homosexual*[Title/ Abstract])) OR (Queer*[Title/Abstract])))) AND ("Randomized Controlled Trial" [Publication Type]).

### Screening criteria

Inclusion criteria: (a) Study type: randomized controlled trial. (b) Study population: sexual and gender minorities with affective symptoms (including but not limited to gay, lesbian, bisexual, transgender, asexual and queer/questioning), regardless of gender identity, race and nationality. (c) Intervention: Psychosocial intervention (intervention by providers including psychologists, psychiatrists, social workers, counselors/therapists, primary care and others). (d) Outcome: affective symptom outcome (including but not limited to depression, anxiety, distress, etc.). (e) Measures: A standardized scale with high reliability and validity should be used for measurement. Exclusion criteria: (a) Reviews, cross-sectional studies, cohort studies, case–control studies, qualitative research, protocols, duplicate literature. (b) Data from the same trial published by the same author. (c) There is no access to the original text. (d) Intervention outcome data were incomplete or could not be combined. The screening was conducted independently by 2 authors, and in the case of dispute, the opinion of another author outside the assessment was sought.

### Data extraction

This study collected data on trial design, primary outcome measures, intervention methods, trial subjects’ age and gender, sample size (trial and control groups), control group treatments, and outcome indicators (posttest and follow-up). For outcomes with continuous variables, mean post-intervention scores and standard deviations of these values were extracted for the trial and control groups, as well as the number of patients included in these analyses. Only the most important intervention group data are included for trials involving multiple interventions. Data were only extracted for the scale used to assess the main outcome indicator when two distinct scales were employed in single research to test the same indicator. The control and experimental data were transformed and pooled if an outcome indicator used a scale where the lower the score, the more severe the symptom. In order to standardize the criteria, only the initial follow-ups are included if there are several follow-up data. All study characteristics and outcome data were extracted independently by two authors, and input was sought from an additional author outside of the assessment in the case of dispute.

### Risk bias assessment

The risk of bias for all included studies was assessed using Review Manager version 5.4, concerning the Cochrane Collaboration criteria for evaluation as follows: (a) Random sequence generation; (b) Allocation concealment; (c) double-blinding of personnel and participants; (d) blinding of outcome assessment; (e) incomplete data; (f) selective reporting; and (g) other biases. Each criterion was rated as "low risk of bias," "high risk of bias," or "unclear risk of bias" [26]. Two writers separately evaluated the bias risk, and in the event of a disagreement, a third author outside of the evaluation was consulted.

### Data analysis

Stata version 16.0 was used for meta-analysis. For the results of the continuous variables after the psychosocial intervention, the standardized mean difference (SMD) of each measurement scale was combined and 95% confidence intervals were used for all results.

Heterogeneity was assessed by the I^2^ statistic, the p-value of the chi-square test for heterogeneity, and visual inspection of the forest plot [27]. When the I^2^ statistic is 0%, it indicates no dispersion, and when the value is large, it indicates a high degree of heterogeneity, where 25% is low heterogeneity, 50% is moderate heterogeneity, and 75% is high heterogeneity [28]. When the I^2^ statistic is greater than 50% or the *p*-value is > 0.10, it indicates a good homogeneity of multiple studies, and a fixed-effects model is used; when the I^2^ statistic is less than 50% or the *p*-value is < 0.10, it indicates significant heterogeneity among studies and a random-effects model is used.

To explore possible sources of heterogeneity, subgroup analyses were conducted on the outcomes included in the study. Subgroup analyses included: the type of psychosocial intervention (cognitive-behavioral therapy, other types); intervention form (online intervention, offline intervention); intervention duration (1–6 weeks, 7–12 weeks); and intervention format (group therapy, individual therapy).

Publication bias was assessed by reviewing contour-enhanced funnel plots [29] and Egger's intercept test [30]. If publication bias was present, the cut-and-patch method [31] was used to adjust for possible bias.

## Results

### Results of the search

**Fig. 1:**
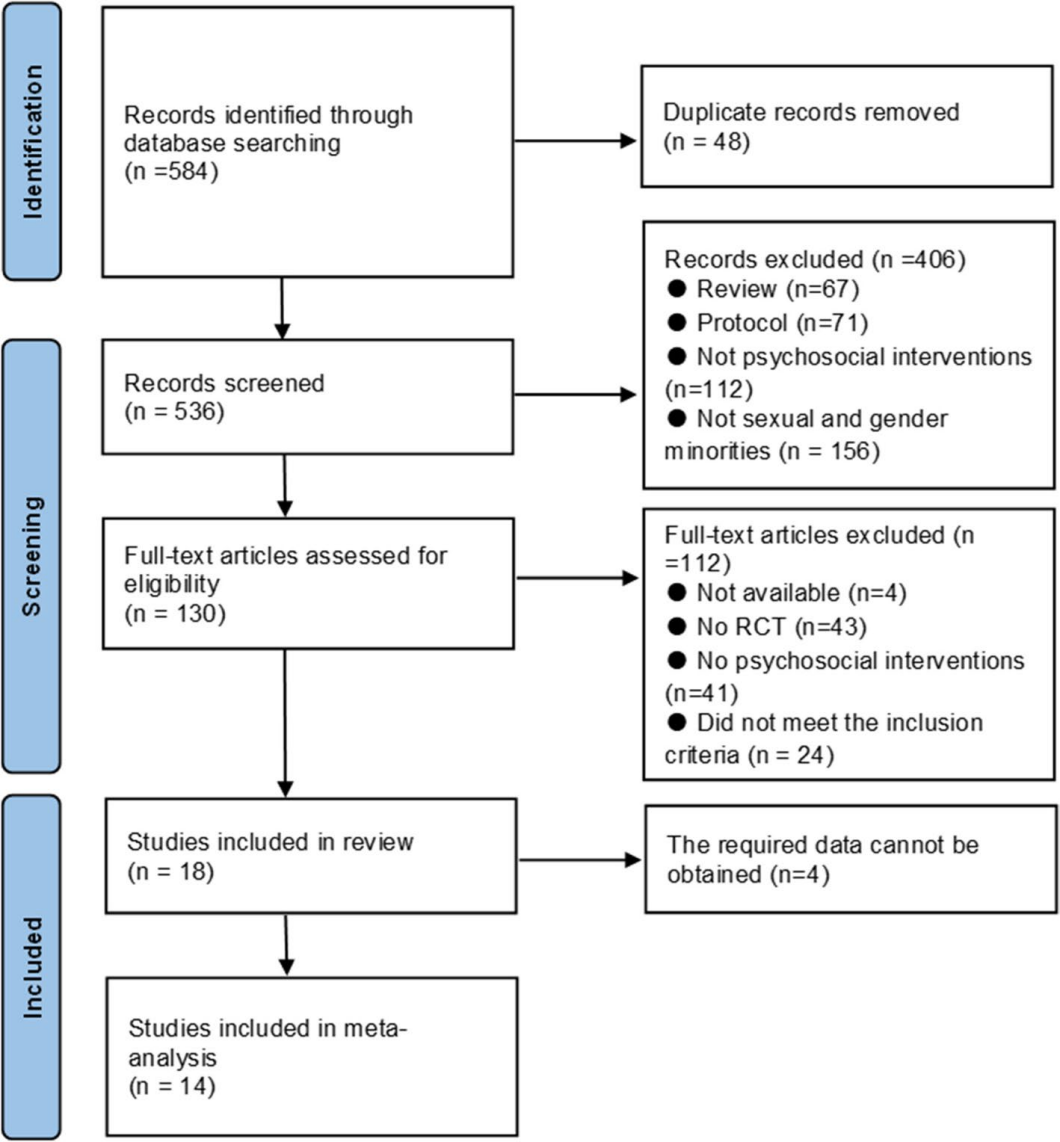
Flow chart of PRISMA study

Searches of the electronic databases resulted in 584 records. After duplicates were removed, there were 536 records. Initial screening excluded 406 records based on title and abstract; 130 records were retrieved and reviewed in full text. Of these studies, a total of 18 met the inclusion criteria. Four studies were missing pre- and post-intervention means, standard deviations, or 95% confidence intervals in the text, tables, or figures, and the authors of those studies did not reply to emails asking these data, leaving 14 studies accessible for preliminary analysis. A PRISMA flow diagram shows the selection of papers for inclusion and exclusion (Fig. 1).

### Study characteristics

Overall, 14 studies included 1103 participants with a sample size of *N* = 40 to 138, mean age of 22.44 to 46.68. For inclusion, 11 studies had participants assigned male at birth, 1 study had participants assigned female at birth, and 2 studies did not impose any gender restrictions. Among these studies, 12 studies measured depressive symptoms,7 studies measured outcome indicators of anxiety symptoms and 3 studies measured distress symptoms. All of the 14 included papers were in English, 11 in the United States, 2 in Canada and 1 in Australia.

In terms of intervention type, 10 (71.43%) were cognitive-behavioral therapy-based intervention techniques, 2 (14.29%) were psychoeducational interventions based on multiple therapeutic approaches, 1 (7.14%) Mindfulness therapy, and 1 (7.14%) writing therapy.

In terms of intervention form, 12 (85.71%) studies used offline interventions; 2 (14.29%) studies used online interventions.

In terms of intervention format, 8 (57.14%) studies used group therapy, and 4 (28.57%) studies used individual therapy.

Regarding the duration of intervention, 12 (85.71%) studies had an intervention duration of 7–12 weeks, and 2 (14.29%) studies had an intervention duration of 1–6 weeks.

In terms of randomized controlled trial settings, 7 (50%) controls were conventional treatment; 7 (50%) study controls were waiting for treatment. 7 (50%) trials had follow-up and 3 of them had no follow-up data.

**Fig. 2:**
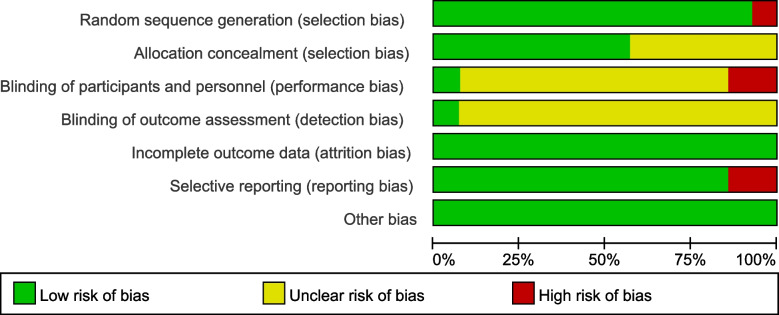
Risk bias assessment chart

Detailed study characteristics are shown in Table 2. The included literature was tested for bias; the results are shown in Fig. 2. The quality assessment of individual study is in appendix 2.

**Table 2:** Characteristics of the included studies

Study (year)	Study setting	Measure	Primary health outcome	Participant mean age (SD/range)	Description of sample (e.g., LGBTIQ status/identification)	Sample size (Experimental group/control group)	Intervention name	Control group	Intervention type
Craig, S. L. (2021) [22]	Canada	BDI-II, PCI-A, RCS, SAMA, HS	Depression, hope, coping, and stress assessment	22.44 (14–29)	Sexual Minority Adolescents	138,97/41	AFFIRM	Waiting	Group intervention, LGB-affirming cognitive behavioral therapy eight times a week for two hours with no follow-up
Pachankis, J.E. (2015) [32]	America	AUDIT, CESD, ODSIS, OASIS, SCS, SSSE, TLFB, MOGS, GRS, IHP, SOCS, DERS, MSPSS, RAS	Depression, anxiety, distress, and co-occurring health risks (i.e., alcohol use, compulsive sexual behavior, condomless sex)	26 (18–35)	Gay, Bisexual Cisgender Male	63,32/31	ESTEEM	Waiting	Individual intervention, LGB-affirming cognitive behavioral therapy, ten sessions, 3-month follow-up
Pachankis, J. E. (2020) [33]	America	CES-D, BSI, ODSIS, OASIS…	Depression, anxiety, distress, minority stress, alcohol use	25.58 (18–35) (3.26)	Sexual Minority Women	60,30/30	EQuIP	Waiting	Individuals, LGB-Affirming Cognitive Behavioral Therapy, 10 weeks
Pachankis, J. E. (2020) [23]	America	CESD, BSI, BAI, SIDAS…	Depression, anxiety, painful alcohol use, suicidal ideation, LGBTQ victimization…	23.68 (18–29) (3.11)	Sexual Minority Adolescents	72,36/36	Expressive writing	Conventional treatment	Online, write once a day for 20 min for three consecutive days, in three stages, with a 3-month follow-up
Antoni, M. H. (2000) [34]	America	HARS, HRSD, DSM-III-R, POMS	Depression, anxiety, and 24-h urinary-free cortisol levels	36.43 (18—55)	gay or bisexual men living with HIV	59,40/19	CBSM	Waiting	Group intervention, 10 weeks
Antoni, M. H. (2000) [35]	America	HARS, HRSD, DSM-III-R, SCID-NP-HIV, POMS, PSS…	Anxiety, perceived stress, 24-h urinary catecholamine levels, and t-lymphocyte subsets	35.97 (18–55)	gay or bisexual men living with HIV	73,47/26	CBSM	Waiting	Group intervention, 10 weeks
Blashill, A. J. (2017) [36]	America	BABS, BIDQ, MADRS, GAF, DSM-IV…	body image disturbance, ART compliance, depression (MADRS), and global functioning (GAF)	46.68 (18–65) (10.51)	gay or bisexual men living with HIV	44,22/22	CBT-BISC	Conventional treatment	Individual intervention with weekly meetings over 3 months (12 phases in total). Each duration lasted approximately 50 min with 6-month follow-up
Carrico, A. W. (2006) [37]	America	POMS, COPE…	Depression, coping strategies, medication adherence…	18–65	gay or bisexual men living with HIV	130,76/54	CBSM	Conventional treatment	CBSM group intervention. Attendance at 135-min sessions per week for ten weeks, with follow-up from June to December
Carrico, A. W. (2005) [24]	America	POM, SPS	Depression, perceived social support, and herpesvirus immunoglobulin G (IgG) antibody titers	34.9 (6.6)	gay or bisexual men living with HIV and on HAART	49,31/18	CBSM	Waiting	CBSM group intervention. Attendance at 135-min sessions per week for ten weeks, with follow-up during 6–12 months
Carrico, A. W. (2005) [38]	America	LES, BDI, SPS, COPE	Depression, perceived social support	35.6(7.2)	gay or bisexual men living with HIV	129,83/46	CBSM	Conventional treatment	CBSM group intervention. Attended weekly 135-min sessions for ten weeks, with a 6-month follow-up
Gayner, B. (2012) [39]	Canada	IES, HADS, PANAS, TMS	Depression, anxiety positive emotions, positive thinking	43.77 (18–70)	gay living with HIV	117,78/39	MBSR	Conventional treatment	Eight 3-h group sessions and one day retreat per week, six days per week for eight weeks. six-month follow-up
Lutgendorf, S. K. (1997) [40]	America	BDI, POMS	Depression, anxiety, physiological indicators	36.75 (20–49)	gay living with HIV	40,22/18	CBSM	Waiting	CBSM group intervention. Attended 135 min of sessions per week for 10 weeks
Millard, T. (2016) [41, 42]	Australia	PROQOL-HIV, heiQ, SF-12, DASS, DSSI, MAH	Pain, quality of life	42.3 (10.4)	gay or MSM living with HIV	132,68/64	Positive Outlook Program	Conventional treatment	online self-management group intervention, participants were encouraged to log into the program for approximately 90 min per week for more than 7 weeks. 12-week follow-up
Williams, J. K. (2013) [43]	America	CSA, PDS, BDI-II	Depression, PTSD, sexual risk behavior…stress biomarkers	46.6(8.3)	African American bisexual men or MSMW living with HIV	88,44/44	ES-HIM	Conventional treatment	6 group sessions lasting 2 h each over 3 weeks with 6-month follow-up

*BDI-II* Beck depression inventory-II. *BCI *Brief COPE Inventory. *PCI-A* Proactive coping inventory for adolescents-A. *RCS *Reflective coping subscale. *SAMA* Stress appraisal measure for adolescents. *HS* Hope scale. *AUDIT* Alcohol Use Disorders Identification. *CESD* Center for Epidemiological Studies Depression Scale. *ODSIS* Overall Depression Severity & Impairment Scale. *OASIS* Overall Anxiety Severity & Impairment Scale. *SCS *Sexual Compulsivity Scale. *SSSE* Safer Sex Self-Efficacy Questionnaire. *TLFB* 90-day Time Line Follow Back. *HRSD* Hamilton Rating Scale for Depression. *POMS* The Profile of Mood States. *TMD* Total Mood Disturbance. *PSS* Perceived Stress Scale. *PROQOL-HIV* HIV-related quality of life. *MADRS* Montgomery-Asberg Depression Rating Scale. *LES* Life Experiences Survey. *PANAS* Positive and Negative Affect Schedule. *IES* The Impact of Event Scale. *DASS* Depression Anxiety and Stress Scale. *DSSI* Duke Social Support Index. *HeiQ *Health Education Impact Questionnaire. *CSA *Childhood sexual abuse. *PDS* Posttraumatic Diagnostic Scale

### Efficacy of psychosocial interventions

#### Depression

**Fig. 3:**
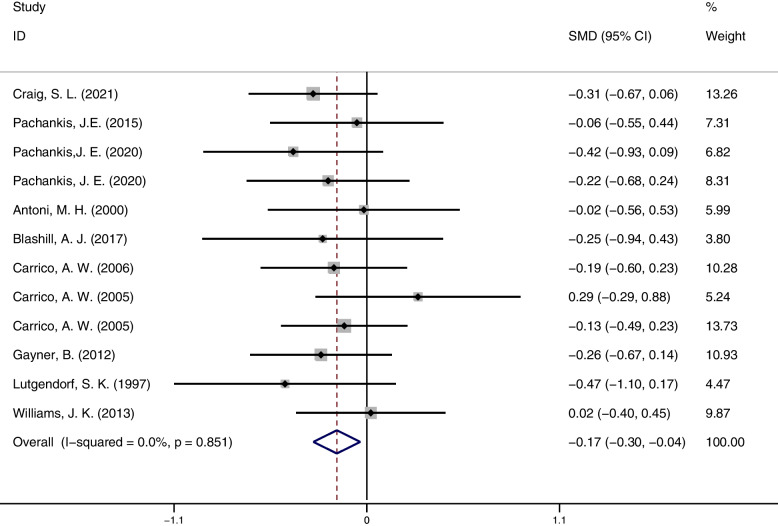
Depression outcome

A meta-analysis of depression outcomes (12 studies, 930 participants) showed that psychosocial interventions were effective in reducing depressive symptoms compared to controls (SMD = -0.17; 95% CI = [-0.30, -0.04]; *p* = 0.012), and studies were more homogeneous (I^2^ = 0.0%; 95% CI = [0.00,49.82]; *p* = 0.851). The forest plots of depression result see Fig. 3.

### Anxiety

**Fig. 4:**
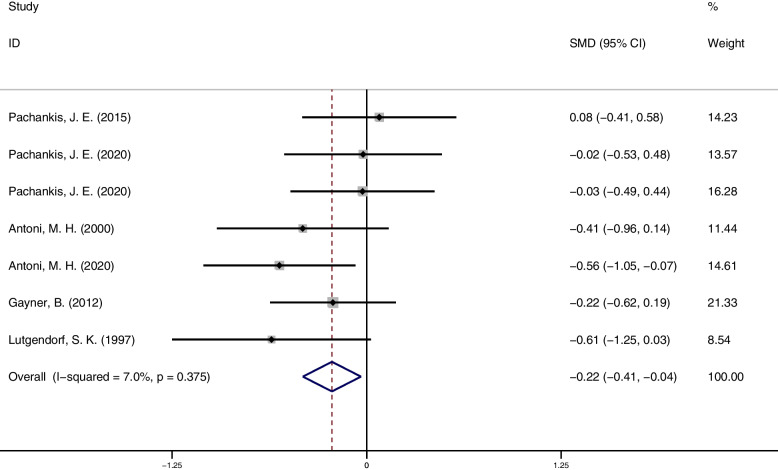
Anxiety outcome

A meta-analysis of anxiety outcomes (7 studies, 195 participants) showed that psychosocial interventions were effective in reducing anxiety symptoms compared to controls (SMD = -0.22; 95%CI = [-0.41, -0.04]; *p* = 0.019), with studies being homogeneous (I^2^ = 7.0%; 95% CI = [0.00,61.30]; *p* = 0.375). The forest plots of anxiety result see Fig. 4.

### Distress

**Fig. 5:**
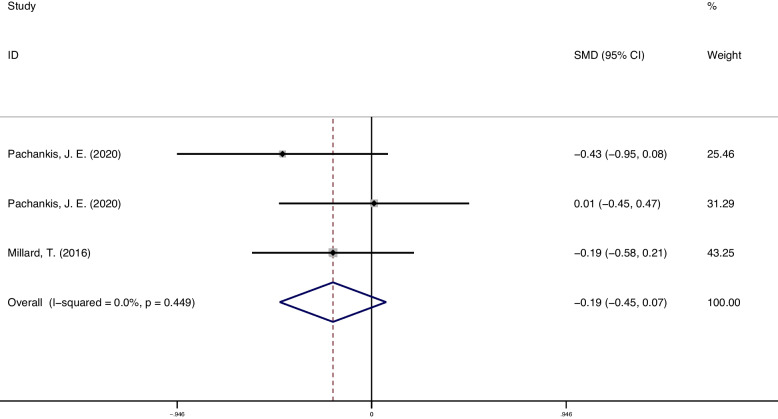
Distress outcome

A meta-analysis of distress outcomes (3 studies, 232 participants) showed that psychosocial interventions were not effective in reducing distress symptoms compared to controls (SMD = -0.19; 95% CI = [-0.45, 0.07]; *p* = 0.021), and studies were homogeneous (I^2^ = 0.0%; 95% CI = [0.00,72.89]; *p* = 0.449). The forest plots of distress result see Fig. 5.

### Trial follow-up

**Fig. 6:**
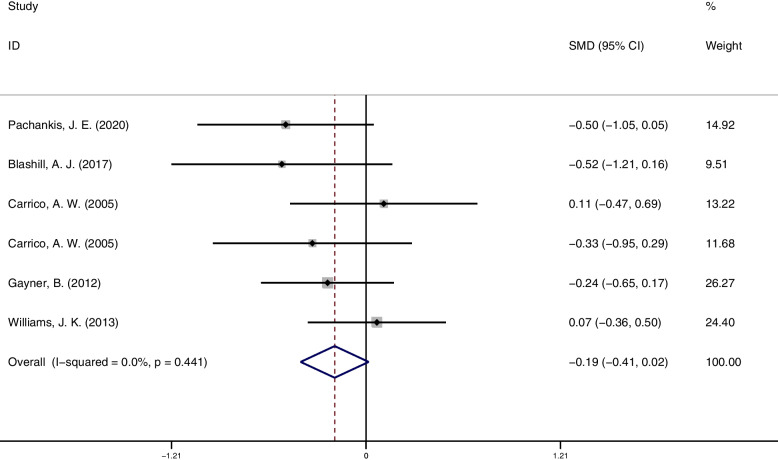
Depression follow-up

The efficacy of psychosocial interventions was not well maintained in the follow-up assessment of depression outcomes (6 studies,360 participants) (SMD = -0.19; 95% CI = [-0.41, 0.02]; *p* = 0.071), and studies were homogeneous (I^2^ = 0.0%; 95% CI = [-0.00. 61.04]; *p* = 0.441). The forest plots of trial follow-up result see Fig. 6.

### Publication bias

Visual inspection of the funnel plot and Egger's linear regression analysis suggested no significant publication bias between studies with depression as an outcome indicator (*p* = 0.735), between studies with anxiety as an outcome indicator (*p* = 0.344), and between studies with distress as an outcome indicator (*p* = 0.250).

### Subgroup analysis

The results of the subgroup analysis are shown in Table 3. Further subgroup analyses were conducted under the two main outcome indicators of depression and anxiety. In terms of intervention duration, 7–12 weeks intervention can alleviate their depression and anxiety symptoms better than the 1–6 weeks intervention. In terms of intervention type, the intervention efficacy using cognitive behavioral therapy was slightly better than other types of interventions in reducing depression and anxiety symptoms. In terms of intervention form, face-to-face intervention alleviates their depression and anxiety symptoms better than online intervention. For anxiety symptoms of SGM, group treatment is more effective than individual treatment.

**Table 3:** Results of subgroup analysis

Outcome measure	Subgroup type	Subgroup	Number of studies	Number of subjects	SMD	95% CI	I^2^	95% CI	*P*
Anxiety	Intervention format	Individual	3	195	0.01	[-0.27,0.29]	0.0%	[0.00,72.89]	0.946
		group	4	275	-0.41	[-0.66, -0.16]	0.0%	[0.00,67.91]	0.001
	Intervention type	CBT/CBSM	5	295	-0.28	[-0.51, -0.04]	28.0%	[0.00,73.26]	0.022
		Others	2	175	-0.13	[-0.44,0.17]	7.0%	\	0.388
	Intervention form	Online	1	72	-0.03	[-0.49,0.44]	\	\	0.911
		Face to face	6	398	-0.26	[-0.46, -0.06]	11.0%	[0.00,65.16]	0.012
	Intervention duration	1–6 weeks	1	72	-0.03	[-0.49,0.44]	\	\	0.911
		7–12 week	6	398	-0.26	[-0.46, -0.06]	11.0%	[0.00,65.16]	0.012
Depression	Intervention type	CBT/CBSM	10	755	-0.15	[-0.30,0.00]	0.0%	[0.00,52.69]	0.043
		Others	2	175	-0.24	[-0.55,0.06]	0.0%	\	0.117
	Intervention duration	1–6 weeks	2	157	-0.09	[-0.40,0.22]	0.0%	\	0.580
		7–12 week	10	773	-0.19	[-0.34, -0.04]	0.0%	[0.00,52.69]	0.012
	Intervention form	Online	1	72	-0.22	[-0.68,0.24]	\	\	0.351
		Face to face	11	858	-0.17	[-0.31, -0.03]	0.0%	[0.00,52.69]	0.019

*CBT *Cognitive behavioral therapy, *CBSM* Cognitive-behavioral stress management

## Discussion

### Main findings

In this study, the effectiveness of psychosocial intervetions on the affective symptoms of SGM was evaluated by a systematic literature search and a meta-analysis of the 14 included publications. The findings demonstrated that psychosocial intervention decreased their symptoms of anxiety and depression. These findings are in line with those of earlier research on psychosocial intervention for anxiety and depression in a variety of populations, such as the reduction of depression in depressed patients with coronary artery disease [44], the enhancement of emotion regulation in young adults [45], and the amelioration of depression in the general public during the COVID-19 pandemic [46].

A CBSM intervention reduced distress symptoms in gay living with HIV [24], and a transdiagnostic minority stress intervention also reduced distress symptoms in sexual minority women [33]. Because expressive writing did not significantly lessen research participants' symptom of distress [23], the combined results of these three trials were not statistically significant. It might also have to do with the few included studies that had insufficient data support. To find more effective psychosocial therapies for SGM's distress symptoms, more research is required. Results from follow-up studies indicated that the reduction of depressive symptoms was not adequately sustained by psychosocial therapies. Psychosocial interventions did not offer long-term efficacy, according to a meta-analysis of these treatments for smoking cessation in individuals with coronary artery disease [47]. Perhaps minority stress processes which lead to distress in SGM, require more time to change [33].

A subgroup analysis was conducted on the factors of psychosocial intervention efficacy. The results showed that face-to-face interventions were more effective than online interventions at reducing depression and anxiety symptoms in SGM. This may be related to lower individual compliance with Internet-based interventions [48]. In terms of intervention format, group therapy was more effective than individual therapy for anxiety symptoms in SGM. Group-based intervention can address the needs of a range of community-based SGMY [22]. This finding is similar to previous studies that group therapy is equally effective as individual treatment for many mental disorders, including anxiety, depression, distress, eating disorders, and schizophrenia [49]. According to previous studies, group therapy has been shown to be effective in reducing symptoms in addition to providing members with a sense of belonging, purpose, hope, altruism, and meaning throughout the treatment process [50]. It can improve social bonding, develop community and personal support, and allow for mutual support and reflection, highlighting the universality of shared experiences [51].

Cognitive behavioral therapy (CBT) is more effective than other therapies for depression and anxiety symptoms in SGM. The efficacy of cognitive-behavioral therapy for adolescent mental health problems has been demonstrated [52, 53]. Affirmative cognitive-behavioral therapy based on CBT and incorporating a theoretical model of minority stress has also been effective for SGM. The most plausible explanation for mental health disparities between SGM and heterosexual groups is the SGM's overexposure to stigma [12, 54]. The sexual minority stress model proposed by Meyer states that minority stress is associated with the experience of stigma and discrimination associated with sexual minority status, which can be extremely stressful for the group and thus contribute to their psychological diseases [11]. The affirmative CBT group intervention (AFFIRM), which positively validates stigmatized identities by acknowledging the impact of interpersonal and structural sources of stigma based on SGM status and targets cognitive, emotional, and behavioral processes, has shown significant improvements in affective symptoms in SGM [22]. The Effective Skills to Empower Effective Men (ESTEEM) significantly reduced depressive symptoms in a sample of gay and bisexual men aged 18–35 by influencing universal factors such as social support and rumination [32]. SGMY-specific intervention techniques target both minority stress processes (i.e., rejection sensitivity, internalized homophobia, concealment) and risk factors prevalent in the shared health status of gay and bisexual men (i.e., despair, rumination, social isolation, lack of self-confidence) [32]. Previous research has also shown that affirmative CBT can effectively address the complex stressors that exacerbate depression and psychological distress in SGMY, help them assess the impact of stress on their well-being, as well as reduce shame-related self-blame and shame [9]. In terms of intervention length, 7–12-week intervention reduced depression and anxiety symptoms in SGM more than 1–6-week intervention, which may be related to the fact that minority stress takes longer to reduce [33]. It shows the importance of choosing a specific, theoretically based and practically integrated CBT for psychosocial interventions in SGM.

### Recommendations for future research

More randomized controlled studies need to be conducted in the future regarding the distressing symptoms of SGM, and more effective interventions are needed for this condition. In addition, more long-term effective interventions for SGM are needed in the future. Finally, there are more interventions for gay and bisexual men than for other populations, and more interventions related to reducing the risk of sexually transmitted infections than other health issues according to the search result. It has also been shown that sexual minority women remain significantly underrepresented in studies of health disparities based on sexual orientation [17], even though they face serious health problems, minority stressors, and more potential additional stressors than SGM men [33]. Therefore, more clinical controlled trials targeted at sexual minority women are needed to explore. From family dysfunction to care inequalities [55, 56], there are many studies on the problems faced by SGM, but there are few randomized controlled trials on how SGM cope with hostility in the environment and how to target psychological interventions for SGM with pre-existing affective symptoms. Thus, more psychosocial interventions are needed for a broader range of health issues, particularly in the area of psychological distress in SGM. In addition, more than half of the studies included in this article were intervention trials for gay living with HIV, but only one of the remaining articles explicitly excluded individuals living with HIV when screening participants. So, while creating subgroups for discussion about heterogeneity, this study did not differentiate between study participants who were living with HIV and those who were not. More cross-sectional studies of SGM living with HIV and without HIV could be conducted in the future to examine how these two groups differ in the effectiveness of psychological therapies.

### Limitations

To begin with, the randomized controlled trials included in this study were uneven in quality. First, some studies did not clearly state the randomization method, blinding, and allocation concealment within 14 studies. Second, it was difficult to determine the long-term efficacy of the psychosocial interventions because five studies did not set follow-up and three studies’ follow-up data were not available. Third, because there were few randomized controlled trials whose search results met the inclusion criteria, the number of articles corresponding to the same outcome index was small. Therefore, to provide more adequate clinical evidence, more high-quality randomized controlled trials with uniform outcome evaluation methods may be needed. Next, Secondly, in terms of data processing, different studies measure the same outcome index in different ways. Four studies use more than one item of scale when measuring the same outcome index. To ensure the rationality of the research results, multiple results were selected. The evaluation criteria have been mentioned above, but there may still be bias in the results. Finally, owing to the limited search strategy, we do not have alternative techniques for database search such as citation search or manual journal search, and the search results may be biased.

## Conclusion

This study found that psychosocial interventions could reduce depression and anxiety symptoms in SGM, but had no significant effect on their psychological distress. The implementation of interventions should be carried out face-to-face group interventions as much as possible, and CBT is currently a more effective intervention. Long-term (7–12 weeks) interventions are more effective than short-term (1–6 weeks). The quality of included studies is limited, more randomized controlled trials with larger sample sizes, multiple follow-up times, and strict study designs should be further conducted to identify the effect of psychosocial intervention on SGM.

### Supplementary Information

**Additional file 1. **

## Data Availability

The datasets used and/or analysed during the current study available from the corresponding author on reasonable request.
