# Supplementary material for: Efficacy of psychosocial interventions to reduce affective symptoms in sexual and gender minorities: a systematic review and meta-analysis of randomized controlled trials
Source: BMC Psychiatry. 2024 Jan 2;24:4. doi: 10.1186/s12888-023-05451-y (PMC10762931; doi:10.1186/s12888-023-05451-y)
Supplement: Supplementary file 1 — Additional file 1. [file 12888_2023_5451_MOESM1_ESM.docx]

appendix 1

| PubMed | ((((("Psychosocial Intervention"[Mesh]) OR ((psychosocial intervention*[Title/Abstract]) OR ( psychological intervention*[Title/Abstract])) OR (("Psychotherapy"[Mesh]) OR (psychotherap*[Title/Abstract])) OR (psychoeducation[Title /Abstract])) AND (("Sexual and Gender Minorities"[Mesh]) OR ((((((((((((((((((Non?Heterosexual*[Title/Abstract]) OR (Sexual Dissident*[Title /Abstract])) OR (GLBT Person*[Title/Abstract])) OR (GLBTQ Person*[Title/Abstract])) OR (LGBT Person*[Title/Abstract])) OR (LGBTQ Person*[Title /Abstract])) OR (Lesbigay Person*[Title/Abstract])) OR (Non?Heterosexual Person*[Title/Abstract])) OR (Sexual Minorit*[Title/Abstract])) OR ( LBG Person*[Title/Abstract])) OR (Gay*[Title/Abstract])) OR (Men Who Have Sex With Men[Title/Abstract])) OR (Gender Minorit*[Title/Abstract])) OR (Lesbian*[Title/Abstract])) OR (Women Who Have Sex With Women[Title/Abstract])) OR (Bisexual*[Title/Abstract])) OR (Homosexual*[Title/Abstract])) OR (Homosexual*[Title/ Abstract])) OR (Queer*[Title/Abstract])))) AND ("Randomized Controlled Trial" [Publication Type]). |
| --- | --- |
| Embase | Title or Abstract=(psychosocial intervention* OR psychological intervention* OR psychotherap*OR psychoeducation) AND Title or Abstract =(Sexual and Gender Minorities OR Non?Heterosexual* OR Sexual Dissident* OR GLBT Person* OR GLBTQ Person* OR LGBT Person* OR LGBTQ Person* OR Lesbigay Person* OR Non?Heterosexual Person* OR Sexual Minorit* OR LBG Person* OR Gay* OR Men Who Have Sex With Men OR Gender Minorit* OR Lesbian* OR Women Who Have Sex With Women OR Bisexual* OR Homosexual* OR Queer*) AND Title or Abstract =(Randomized Controlled Trial) |
| Cochrane Library | (psychosocial intervention*):ti,ab,kw (Word variations have been searched) OR (psychological intervention* OR psychotherap*OR psychoeducation):ti,ab,kw (Word variations have been searched) AND (Sexual and Gender Minorities):ti,ab,kw (Word variations have been searched) OR (Non?Heterosexual* OR Sexual Dissident* OR GLBT Person* OR GLBTQ Person* OR LGBT Person* OR LGBTQ Person* OR Lesbigay Person* OR Non?Heterosexual Person* OR Sexual Minorit* OR LBG Person* OR Gay* OR Men Who Have Sex With Men OR Gender Minorit* OR Lesbian* OR Women Who Have Sex With Women OR Bisexual* OR Homosexual* OR Queer*):ti,ab,kw (Word variations have been searched) AND (Randomized Controlled Trial):ti,ab,kw (Word variations have been searched) |
| CNKI | （主题：性少数群体 + 同性恋+ 双性恋 + 跨性别（精确）） AND (主题：社会心理干预 + 社会干预 + 心理干预 + 心理治疗（精确）) AND （主题：随机对照实验） |
| Wanfang Data | （主题词扩展）： 题名或关键词:("性少数群体 OR 同性恋 OR 双性恋 OR 跨性别") and 题名或关键词:("社会心理干预 OR 社会干预 OR 心理干预 OR 心理治疗") and 题名或关键词:(随机对照试验) |
| Web of Science | TS=(psychosocial intervention* OR psychological intervention* OR psychotherap*OR psychoeducation) AND TS=(Sexual and Gender Minorities OR Non?Heterosexual* OR Sexual Dissident* OR GLBT Person* OR GLBTQ Person* OR LGBT Person* OR LGBTQ Person* OR Lesbigay Person* OR Non?Heterosexual Person* OR Sexual Minorit* OR LBG Person* OR Gay* OR Men Who Have Sex With Men OR Gender Minorit* OR Lesbian* OR Women Who Have Sex With Women OR Bisexual* OR Homosexual* OR Queer*) AND TS=(Randomized Controlled Trial) |
| PsycINFO (APA PsycNet) | Any Field: psychosocial intervention AND Any Field: Sexual and Gender Minorities AND Any Field: Randomized Controlled Trial |

appendix 2

| **Study** | **Random sequence generation** | **Allocation concealment** | **Blinding of participants and personnel** | **Blinding of outcome assessment** | **Incomplete outcome data** | **Selective reporting** | **Other bias(Executive intervention)** |
| --- | --- | --- | --- | --- | --- | --- | --- |
| Craig, S.L. (2021) | 3 | 2 | 1 | 2 | 1 | 1 | 1 |
| Pachankis, J.E.（2015） | 1 | 2 | 2 | 2 | 1 | 1 | 1 |
| Pachankis, J.E.(2020) | 1 | 1 | 2 | 2 | 1 | 3 | 1 |
| Pachankis, J.E.(2020)（2） | 1 | 1 | 2 | 2 | 1 | 1 | 1 |
| Antoni, M. H.（2000） | 1 | 1 | 2 | 2 | 1 | 1 | 1 |
| Antoni, M. H.（2000）（2） | 1 | 1 | 2 | 2 | 1 | 1 | 1 |
| Blashill, A. J.（2017） | 1 | 1 | 2 | 2 | 1 | 1 | 1 |
| Carrico, A. W.（2006） | 1 | 1 | 3 | 1 | 1 | 1 | 1 |
| Carrico, A. W.（2005） | 1 | 2 | 2 | 2 | 1 | 1 | 1 |
| Carrico, A. W.（2005）（2） | 1 | 2 | 2 | 2 | 1 | 1 | 1 |
| Gayner, B.（2012） | 1 | 1 | 2 | 2 | 1 | 1 | 1 |
| Lutgendorf, S. K.（1997） | 1 | 2 | 2 | 2 | 1 | 1 | 1 |
| Millard, T.（2016） | 1 | 1 | 3 | 2 | 1 | 3 | 1 |
| Williams, J. K.（2013） | 1 | 2 | 2 | 2 | 1 | 1 | 1 |
| Cochrane：1 Low、2 Unclear、3 High | | | | | | | |
